# Clinical Implications of the Autophagy Core Gene Variations in Advanced Lung Adenocarcinoma Treated with Gefitinib

**DOI:** 10.1038/s41598-017-18165-5

**Published:** 2017-12-19

**Authors:** Jupeng Yuan, Nasha Zhang, Longbin Yin, Hui Zhu, Li Zhang, Liqing Zhou, Ming Yang

**Affiliations:** 1 Shandong Provincial Key Laboratory of Radiation Oncology, Cancer Research Center, Shandong Cancer Hospital affiliated to Shandong University, Shandong Academy of Medical Sciences, Jinan, Shandong Province China; 2 Department of Radiation Oncology, Shandong Cancer Hospital affiliated to Shandong University, Shandong Academy of Medical Sciences, Jinan, Shandong Province China; 3 Department of Oncology, Tongji Hospital, Tongji Medical College, Huazhong University of Science and Technology, Wuhan, Hubei Province China; 4 Department of Radiation Oncology, Huaian No. 2 Hospital, Huaian, Jiangsu Province China

## Abstract

EGFR-TKIs show dramatic treatment benefits for advanced lung adenocarcinoma patients with activating *EGFR* mutations. Considering the essential role of autophagy in EGFR-TKIs treatments, we hypothesized that genetic variants in autophagy core genes might contribute to outcomes of advanced lung adenocarcinoma treated with gefitinib. We systematically examined 27 potentially functional genetic polymorphisms in 11 autophagy core genes among 108 gefitinib-treated advanced lung adenocarcinoma patients. We found that *ATG10* rs10036653, *ATG12* rs26538, *ATG16L1* rs2241880 and *ATG16L2* rs11235604 were significantly associated with survival of lung adenocarcinoma patients (all *P* < 0.05). Among *EGFR*-mutant patients, *ATG5* rs688810, *ATG5* rs510432, *ATG7* rs8154, *ATG10* rs10036653, *ATG12* rs26538, *ATG16L1* rs2241880 and *ATG16L2* rs11235604 significantly contributed to disease prognosis. We also found that *ATG5* rs510432, *ATG5* rs688810, *ATG10* rs10036653 and *ATG10* rs1864182 were associated with primary or acquired resistance to gefitinib. Functional analyses of *ATG10* rs10036653 polymorphism suggested that *ATG10* A allele might increase transcription factor OCT4 binding affinity compared to the T allele in lung cancer cells. Our results indicate that autophagy core genetic variants show potential clinical implications in gefitinib treatment, especially among advanced lung adenocarcinoma patients, highlighting the possibility of patient-tailored decisions during EGFR-TKIs based on both germline and somatic variation detection.

## Introduction

Lung cancer is one of most common and lethal cancers worldwide. Currently, it is classified to two major pathological types^1^. About 80% of lung cancer patients are characterized as non-small cell lung cancer (NSCLC) and 20% as small cell lung cancer (SCLC). For NSCLC, there are several subtypes, such as adenocarcinoma, squamous cell carcinoma, and large cell carcinoma, and all types can occur in unusual histologic variants^2^. NSCLC are relatively insensitive to chemotherapy and/or radiotherapy compared to SCLC^2^. Epidermal growth factor receptor (EGFR) with activating mutations has been proved to be a promising therapeutic target of EGFR tyrosine kinase inhibitors (EGFR-TKIs) for NSCLC^3,4^. Compared with platinum-based chemotherapy, EGFR-TKIs show great advantages by significantly prolonging progression-free survival (PFS)^5^. Advanced NSCLC patients, especially ones with adenocarcinoma histology and *EGFR* active mutations, show great clinical benefits from EGFR-TKIs^6^. The frequency of *EGFR* mutations are highest in East Asia populations including Chinese^7–9^. However, most patients, even cases with *EGFR* mutations, develop drug resistance after a median PFS of 10–16 months, followed by disease progression after initial EGFR-TKIs treatment^10^. The detailed mechanisms responsible for EGFR-TKIs resistance are still not fully understood, which greatly limited their application in clinic.

Autophagy is an evolutionarily conserved process which is essential for survival, differentiation, development, and homeostasis. As a lysosomal degradation pathway, autophagy can maintain cell homeostasis through degrading damaged organelles and long-lived proteins^11,12^. It has been reported that autophagy is involved in multiple diseases, for example cancers, infections, neurodegeneration and aging^13–16^. During cancer development, autophagy is considered as a non-apoptotic cell death pathway and suppresses tumorigenesis under certain circumstances. However, autophagy facilitates tumorigenesis in most contexts^17–19^. Autophagosome is a kind of spherical organelle with double layer membranes during autophagy. Establishment of autophagosome is controlled by several autophagy core genes^20^, which might be involved in cancer initiation and progression^21^.

Accumulating evidences indicate that germline genetic variants may also play a part in resistance to EGFR-TKIs. For instance, Ng *et al*. reported that NSCLC patients harboring *EGFR* mutations showed better clinical response to TKIs if the patients carried a germline deletion polymorphism in *BCL2L11* (*BIM*) at the same time^22^. Moreover, we also found that *EGFR* germline polymorphisms (rs2293347 and rs4947492) might be potential predictive markers of overall survival (OS) in advanced lung adenocarcinoma patients treated with gefitinib^23^. In the current study, we hypothesized that genetic variants of autophagy core genes may contribute to differential prognostic outcomes of advanced lung adenocarcinoma patients treated with gefitinib. To address this, we systematically examined the clinical implications of 23 potentially functional polymorphisms in ten autophagy core genes (*ATG2B*, *ATG3*, *ATG4C*, *ATG5*, *ATG7*, *ATG9B*, *ATG10*, *ATG12*, *ATG16L2* and *BECN*) in advanced lung adenocarcinoma who received gefitinib therapy.

## Materials and Methods

### Study subjects

There is a total of 108 patients with advanced lung adenocarcinoma treated with gefitinib in this study (Supplementary Table 1). Patients were recruited between July 2003 and July 2012 at Tongji Hospital, Tongji Medical College, Huazhong University of Science and Technology (Wuhan, Hubei Province, China). As reported previously, eligible patients had at least one measurable lesion with a minimum size in at least one diameter of ≥10 mm for liver, lung, brain or lymph node metastases, WHO performance status of 0-1, and life expectancy of ≥3 months^23^. Each patient was treated with gefitinib orally at a daily dose of 250 mg as 2nd or 3rd line monotherapy. The exclusion criteria included previous other EGFR-TKIs treatment, pneumonectomy or severe cardio-pulmonary diseases^23^. This study was approved by the Review Boards of Tongji Hospital, Tongji Medical College and Shandong Cancer Hospital affiliated to Shandong University. Written informed consent from each patient for the use of his/her DNA and clinical information was obtained. The methods were carried out in accordance with the approved guidelines.

### Genetic variants selection of autophagy core genes

Single nucleotide polymorphisms (SNPs) of autophagy core genes were selected as previously described^24^. In briefly, common SNPs (MAF ≥ 0.05 in Chinese Han population) in eleven autophagy core genes (*ATG2B*, *ATG3*, *ATG4C*, *ATG5*, *ATG7*, *ATG9B*, *ATG10*, *ATG12*, *ATG16L1*, *ATG16L2* and *BECN*) were screened in the gene regions including a 10-kb up-stream region of each gene based on the HapMap database. A total of 27 potentially functional SNPs were finally selected according to linkage disequilibrium (LD) analyses with an *r* ^2^ threshold of 0.80 as well as prediction with SNPinfo Web Server (http://snpinfo.niehs.nih.gov/).

### Genotyping

Genomic DNA was extracted from blood sample which was collected from each patient upon recruitment. The *ATG3* rs2705507 polymorphism was excluded from the 27 SNPs since it cannot be analyzed by the MassArray system (Sequenom Inc., San Diego, California, USA). The other 26 SNPs were finally determined to be genotyped as described previously^25–28^. However, *BECN* rs9890617, rs9891429 and rs10512488 were excluded because of genotyping failure. As a result, a total of 23 SNPs were successfully genotyped. A 15% blind, random samples were genotyped in duplicates and the reproducibility was 100%.

### Quantitative reverse transcription PCR (qRT-PCR)

After lung cancer A549 cells were transfected with siRNAs of *OCT4*, *MTF1* or *SOX5* (Supplementary Table 2), total RNA was isolated from cells with Trizol reagent (Invitrogen) and treated with RNase-Free DNase to remove genomic DNA (Invitrogen). These RNA samples were then reverse transcribed into cDNAs using Revert Ace kit (TOYOBO, Osaka, Japan). *OCT4*, *MTF1*, *SOX5 ATG5*, *ATG10* and *β-actin* mRNAs were measured through the SYBR-Green qRT-PCR. The *OCT4*, *MTF1*, *SOX5 ATG5* or *ATG10* expression was calculated relative to the *β-actin* expression.

### Electrophoretic Mobility-Shift Assays (EMSA)

Synthetic double-stranded and 3′ biotin-labeled oligonucleotides corresponding to the *ATG10* rs10036653T or rs10036653A sequences (Supplementary Table 2) and A549 cell nuclear extracts were incubated at 25 °C for 20 min using the Light Shift Chemiluminescent EMSA Kit (Pierce, Rockford, IL). The reaction mixture was separated on 6% PAGE, and the products were detected by Stabilized Streptavidin-Horseradish Peroxidase Conjugate (Pierce).

### Statistics

The differences in patients’ characteristics were assessed by Pearson’s χ^2^ tests or Student’s *t* test. Univariate and multivariate Cox proportional hazard regression analyses were utilized to calculate hazard ratios (HRs) and 95% confidence intervals (CIs). Sex, age, smoking status, ECGO and stages were used as adjustment factors during multivariate analyses. Survival differences were examined using the log-rank test. *P* values less than 0.05 were considered significant. All *P* values represent two-sided statistical tests. All statistical procedures were conducted using the SPSS software (version 19.0) and GraphPad Prism7.

## Results

### Advanced lung adenocarcinoma patients’ characteristics and clinical outcomes

As shown in Supplementary Table 1, the distribution of demographic and clinical characteristics of patients were summarized. A total of 108 advanced lung adenocarcinoma patients were enrolled in this study. All individuals were ethnic Han Chinese. The mean age of subjects was 56 ranging from 46 to 66. There were 53 males and 55 females. All the patients were treated with gefitinib and 75% patients were detected with *EGFR* mutations. By the time of the final analysis, the median follow-up time was 29.0 months, and the median PFS and OS was 12.4 and 24.4 months, respectively.

### Effects the autophagy core gene SNPs on PFS and OS of gefitinib-treated advanced lung adenocarcinoma patients

The detailed information of selected 26 autophagy core gene SNPs were summarized in Supplementary Table 3. A total of 23 potentially functional SNPs from 11 autophagy core genes were successfully genotyped. These SNPs were located in introns, exons, 3′ UTR and promoters of autophagy core genes. Associations between the 23 SNPs and PFS or OS were examined using multivariate Cox regression analyses among all patients as well as patients with *EGFR* mutations. As shown in Table 1, *ATG10* rs10036653, *ATG12* rs26538, *ATG16L1* rs2241880 and *ATG16L2* rs11235604 were significantly associated with OS of gefitinib-treated advanced lung adenocarcinoma patients (all *P* < 0.05). *ATG12* rs26538 TT genotype also significantly contributed to increased risk of shorten PFS (HR = 2.47, 95% CI = 1.15-5.31, *P* = 0.021). In the stratified analyses, *ATG5* rs510432 A allele, *ATG7* rs8154 T allele and *ATG10* rs10036653 C allele were proved to be protective alleles which were significantly associated with good prognosis of patients with *EGFR* mutations (PFS: HR = 0.55, 95% CI = 0.32-0.92, *P = *0.022 for *ATG5* rs510432; HR = 0.57, 95% CI = 0.33-0.99, *P = *0.045 for *ATG7* rs8154; HR = 0.48, 95% CI = 0.29-0.79, *P = *0.004 for *ATG10* rs10036653; OS: HR = 0.61, 95% CI = 0.37-1.00, *P = *0.050; HR = 0.56, 95% CI = 0.32-0.96, *P = *0.034; HR = 0.46, 95% CI = 0.27-0.76, *P = *0.003). *ATG5* rs688810, *ATG12* rs26538, *ATG16L1* rs2241880 and *ATG16L2* rs11235604 were risk SNPs whose minor alleles were significantly associated with bad prognosis of patients with *EGFR* mutations (PFS: HR = 1.83, 95% CI = 1.08-3.08 for *ATG5* rs688810, *P = *0.025; HR = 2.51, 95% CI = 1.00-6.30, *P = *0.049 for *ATG12* rs26538; HR = 1.64, 95% CI = 1.00-2.68, *P = *0.050 for *ATG16L1* rs2241880; HR = 1.92, 95% CI = 1.04-3.55, *P = *0.036 for *ATG16L2* rs11235604; OS: HR = 1.76, 95% CI = 1.06-2.91, *P = *0.028; HR = 3.17, 95% CI = 1.22-8.23, *P = *0.018; HR = 1.72, 95% CI = 1.06-2.79, *P = *0.027; HR = 2.28, 95% CI = 1.24-4.22, *P = *0.008). However, other genetic variants of autophagy core genes did not significantly affect PFS or OS (all *P* > 0.05) (Supplementary Table 4 and Supplementary Table 5).

**Table 1:** Associations of genetic variants of autophagy core genes with OS and PFS of advanced lung adenocarcinoma patients treated with gefitinib.

Genes	SNPs	Genotypes	Patients No. (%)	OS		PFS	
				HR (95% CI)	*P*	HR (95% CI)	*P*
*ATG5*	rs510432		105				
		GG	43(40.95)	Reference		Reference	
		GA	41(39.05)	0.72 (0.44-1.17)	0.180	0.79 (0.48-1.31)	0.362
		AA	21(20.00)	0.63 (0.34-1.15)	0.129	0.64 (0.34-1.19)	0.157
		GA + AA	62(59.05)	0.73 (0.48-1.12)	0.152	0.79 (0.50-1.23)	0.290
*ATG5*	rs688810		106				
		TT	41(38.68)	Reference		Reference	
		TC	43(40.57)	1.14 (0.71-1.82)	0.590	1.21 (0.74-1.98)	0.446
		CC	22(20.75)	1.44 (0.83-2.50)	0.191	1.36 (0.77-2.40)	0.289
		TC + CC	65(61.32)	1.30 (0.85-1.98)	0.238	1.31 (0.85-2.04)	0.226
*ATG7*	rs8154		108				
		TT	69(63.89)	Reference		Reference	
		TC	37(34.26)	0.76 (0.49-1.18)	0.220	0.71 (0.45-1.14)	0.155
		CC	2(1.85)	1.55 (0.30-8.02)	0.600	1.98 (0.37-10.51)	0.425
		TC + CC	39(36.11)	0.77 (0.51-1.18)	0.235	0.75 (0.48-1.17)	0.200
*ATG10*	rs10036653		107				
		AA	40(37.38)	Reference		Reference	
		AT	52(48.60)	0.70 (0.43-1.11)	0.130	0.66 (0.40-1.07)	0.090
		TT	15(14.02)	0.43 (0.21-0.89)	0.022	0.56 (0.27-1.14)	0.108
		AT + TT	67(62.62)	0.67 (0.43-1.03)	0.068	0.66 (0.43-1.04)	0.074
*ATG12*	rs26538		107				
		CC	40(37.38)	Reference		Reference	
		CT	54(50.47)	1.08 (0.69-1.70)	0.738	0.99 (0.62-1.57)	0.966
		TT	13(12.15)	2.83 (1.31-6.15)	0.008	2.47 (1.15-5.31)	0.021
		CT + TT	67(62.62)	1.19 (0.079-1.81)	0.404	1.12 (0.73-1.71)	0.608
*ATG16L1*	rs2241880		106				
		TT	44(41.51)	Reference		Reference	
		TC	44(41.51)	1.63 (1.01-2.61)	0.044	1.42 (0.87-2.32)	0.158
		CC	18(16.98)	1.62 (0.85-3.08)	0.142	1.69 (0.89-3.22)	0.109
		TC + CC	62(58.49)	1.62 (1.05-2.49)	0.029	1.42 (0.92-2.20)	0.118
*ATG16L2*	rs11235604		108				
		CC	89(82.41)	Reference		Reference	
		CT	18(16.67)	1.78 (1.07-2.96)	0.028	1.54 (0.93-2.56)	0.094
		TT	1(0.93)	N.C.	N.C.	N.C.	N.C.
		CT + TT	19(17.60)	1.83 (1.11-3.02)	0.018	1.59 (0.97-2.61)	0.068
Genes	SNPs	Genotypes	Patients with *EGFR* mutation No. (%)	OS of patients with *EGFR* mutations		PFS of patients with *EGFR* mutations	
				HR (95% CI)	*P*	HR (95% CI)	*P*
*ATG5*	rs510432		78				
		GG	33(42.31)	Reference		Reference	
		GA	28(35.90)	0.64 (0.36-1.15)	0.136	0.59 (0.32-1.09)	0.094
		AA	17(21.79)	0.58 (0.30-1.15)	0.118	0.47 (0.23-0.96)	0.038
		GA + AA	45(57.69)	0.61 (0.37-1.00)	0.050	0.55 (0.32-0.92)	0.022
*ATG5*	rs688810		80				
		TT	31(38.75)	Reference		Reference	
		TC	31(38.75)	1.49 (0.84-2.64)	0.172	1.58 (0.87-2.89)	0.135
		CC	18(22.50)	2.03 (1.09-3.79)	0.026	1.98 (1.04-3.77)	0.039
		TC + CC	49(61.25)	1.76 (1.06-2.91)	0.028	1.83 (1.08-3.08)	0.025
*ATG7*	rs8154		81				
		TT	55(67.90)	Reference		Reference	
		TC	26(32.50)	0.56 (0.32-0.96)	0.034	0.57 (0.33-0.99)	0.045
		CC	0(0.00)	N.C.	N.C.	N.C.	N.C.
		TC + CC	26(32.50)	0.56 (0.32-0.96)	0.034	0.57 (0.33-0.99)	0.045
*ATG10*	rs10036653		80				
		AA	28(35.00)	Reference		Reference	
		AT	43(53.75)	0.49 (0.29-0.84)	0.009	0.51 (0.30-0.86)	0.012
		TT	9(11.25)	0.23 (0.08-0.87)	0.007	0.39 (0.15-1.03)	0.057
		AT + TT	52(65.00)	0.46 (0.27-0.76)	0.003	0.48 (0.29-0.79)	0.004
*ATG12*	rs26538		80				
		CC	31(38.75)	Reference		Reference	
		CT	40(50.00)	0.95 (0.56-1.82)	0.858	1.00 (0.58-1.71)	0.991
		TT	9(11.25)	3.17 (1.22-8.23)	0.018	2.51 (1.00-6.30)	0.049
		CT + TT	49(61.25)	1.12 (0.69-1.82)	0.657	1.14 (0.70-1.86)	0.608
*ATG16L1*	rs2241880		80				
		TT	36(45.00)	Reference		Reference	
		TC	31(38.75)	1.83 (1.07-3.13)	0.027	1.76 (1.02-3.03)	0.044
		CC	13(16.25)	1.65 (0.81-3.35)	0.168	1.68 (0.82-3.42)	0.156
		TC + CC	44(55.00)	1.72 (1.06-2.79)	0.027	1.64 (1.00-2.68)	0.050
*ATG16L2*	rs11235604		81				
		CC	66(81.48)	Reference		Reference	
		CT	14(17.28)	2.24 (1.20-4.19)	0.012	1.88 (1.01-3.52)	0.047
		TT	1(1.23)	N.C.	N.C.	N.C.	N.C.
		CT + TT	15(18.51)	2.28 (1.24-4.22)	0.008	1.92 (1.04-3.55)	0.036

Note: PFS, progression-free survival; OS, overall survival; HR, hazard ratio; CI, confidence interval; N.C., not calculated.

Hazard ratios (HRs) and 95% confidence intervals (CIs) for the association between SNPs and PFS as well as OS were estimated by Cox regression adjusted by sex, age, smoking status, ECGO and stages.

**Figure 1:**
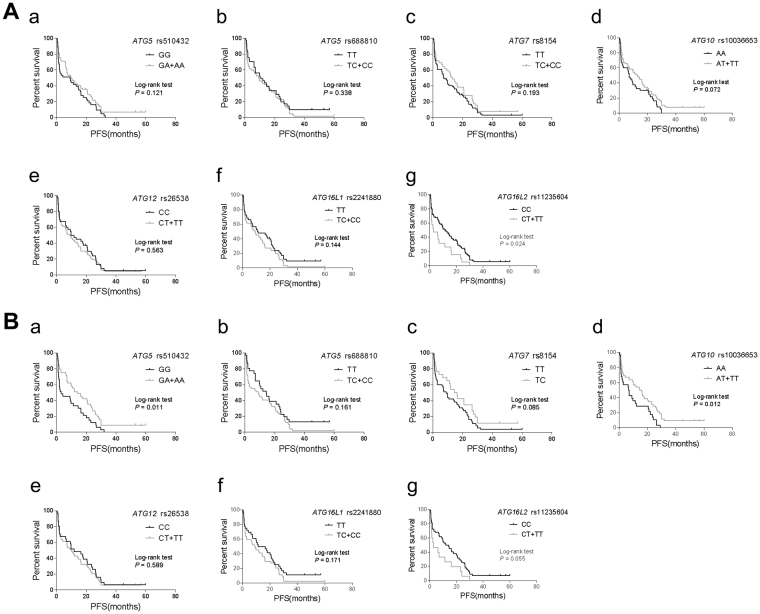
Kaplan-Meier curves of PFS for advanced lung adenocarcinoma patients treated with gefitinib. (**A**) Kaplan-Meier curves of PFS for all NSCLC patients harboring *ATG5* rs510432 (**a**), *ATG5* rs6888810 (**b**), *ATG7* rs8154 (**c**), *ATG10* rs10036653 (**d**), *ATG12* rs26538 (**e**), *ATG16L1* rs2241880 (**f**) and *ATG16L2* rs11235604 (**g**). (**B**) Kaplan-Meier curves of PFS for *EGFR* mutant NSCLC patients harboring *ATG5* rs510432 (**a**), *ATG5* rs6888810 (**b**), *ATG7* rs8154 (**c**), *ATG10* rs10036653 (**d**), *ATG12* rs26538 (**e**), *ATG16L1* rs2241880 (**f**) and *ATG16L2* rs11235604 (**g**). Long-rank analysis was performed, and *P* values less than 0.05 were considered significant.

**Figure 2:**
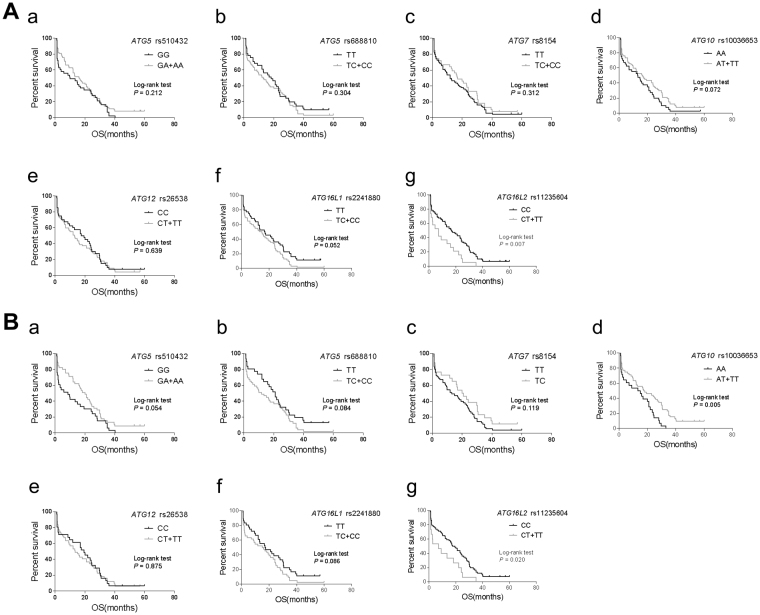
Kaplan-Meier curves of OS for advanced lung adenocarcinoma patients treated with gefitinib. (**A**) Kaplan-Meier curves of OS for all NSCLC patients harboring *ATG5* rs510432 (**a**), *ATG5* rs6888810 (**b**), *ATG7* rs8154 (**c**), *ATG10* rs10036653 (**d**), *ATG12* rs26538 (**e**), *ATG16L1* rs2241880 (**f**) and *ATG16L2* rs11235604 (**g**). (**B**) Kaplan-Meier curves of OS for *EGFR* mutant NSCLC patients harboring *ATG5* rs510432 (**a**), *ATG5* rs6888810 (**b**), *ATG7* rs8154 (**c**), *ATG10* rs10036653 (**d**), *ATG12* rs26538 (**e**), *ATG16L1* rs2241880 (**f**) and *ATG16L2* rs11235604 (**g**). Long-rank analysis was performed, and *P* values less than 0.05 were considered significant.

We also compared the PFS or OS of patients with different genotypes of the aforementioned seven autophagy SNPs (Fig. 1 and Fig. 2). Log-rank tests demonstrated that patients harboring *ATG16L2* rs11235604 T allele had significantly shorten PFS compared to the C allele (3.0 months vs 11.8 months, *P* = 0.024) (Fig. 1A). Among *EGFR* mutant patients, *ATG5* rs510432 A allele or *ATG10* rs10036653 T allele showed significantly prolonged PFS compared to *ATG5* rs510432 G allele or *ATG10* rs10036653 A allele (4.37 months vs 3.07 months, *P* = 0.011 for *ATG5* rs510432; 15.37 months vs 6.82 months, *P* = 0.012 for *ATG10* rs10036653) (Fig. 1B). Similarly, carriers of *ATG16L2* rs11235604 T allele had significantly shorten OS after gefitinib treatment compared to the C allele (7.1 months vs 17.0 months, *P* = 0.007 for all patients; 7.1 months vs 18.4 months, *P* = 0.020 for *EGFR* mutant patients) (Fig. 2A,B). Also, *EGFR* mutant patients with *ATG10* rs10036653 T allele showed significantly prolonged OS compared to the A allele (17.85 months vs 14.18 months, *P* = 0.005) (Fig. 2B). These results elucidated that *ATG5* rs510432, *ATG10* rs10036653 and *ATG16L2* rs11235604 germline polymorphisms might be independent prognostic marker of gefitinib treatment besides somatic *EGFR* mutations.

### Impacts of autophagy core gene SNPs on gefitinib-resistance

**Figure 3:**
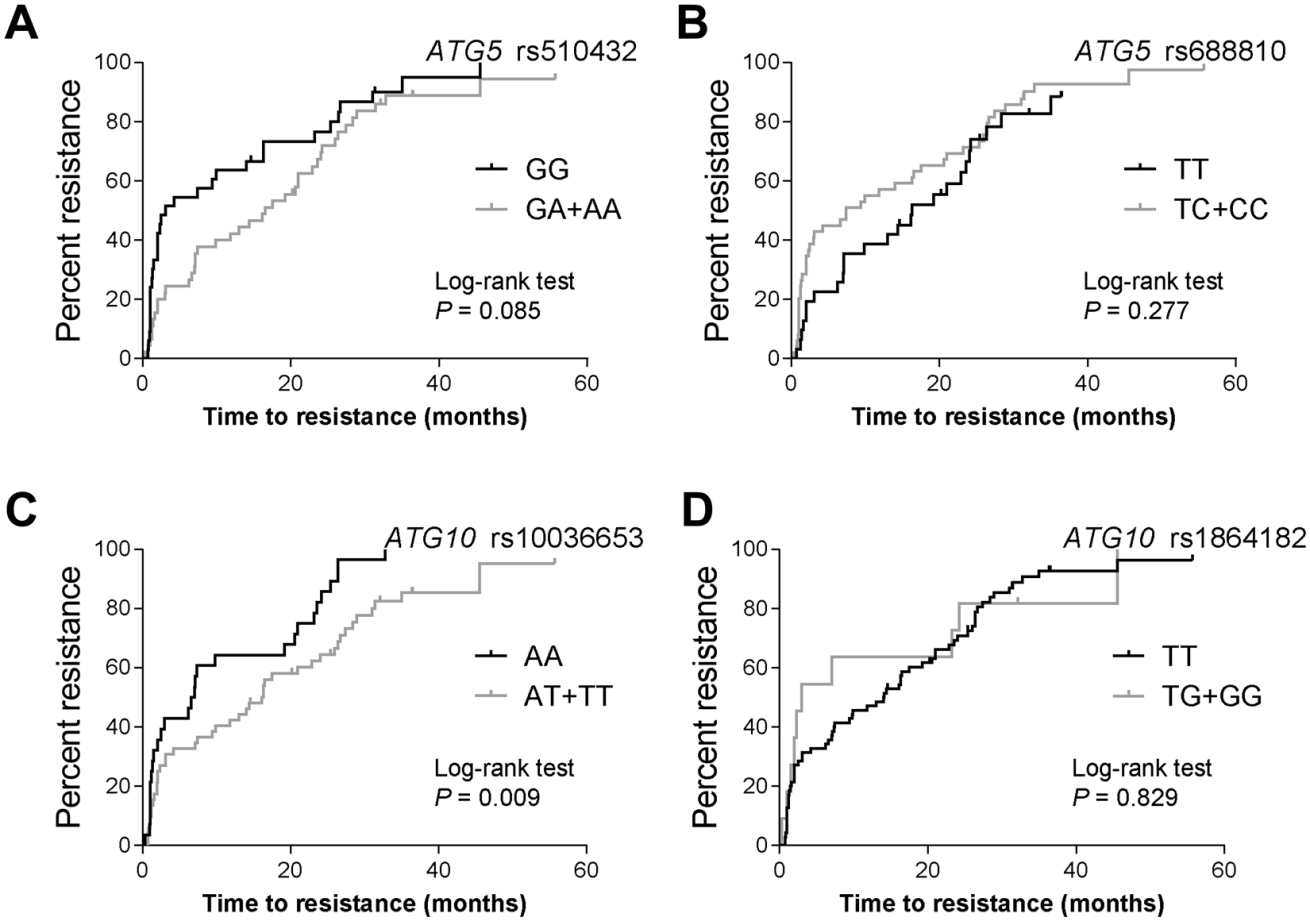
Gefitinib-resistance for *EGFR* mutant lung adenocarcinoma patients harboring different genotypes of autophagy core gene genes. (**A**) *ATG5* rs510432, (**B**) *ATG5* rs688810, (**C**) *ATG10* rs10036653, (**D**) *ATG10* rs1864182. *P* values less than 0.05 were considered significant.

Drug resistance to EGFR-TKIs inevitably develops after a period of effective drug treatment. Here we investigated whether autophagy core gene SNPs could be used as reasonable biomarkers for gefitinib-resistance in advanced lung adenocarcinoma. As shown in Table 2, *ATG5* rs510432 acts as a protective SNP significantly associated with 55% decreased risk of primary gefitinib resistance (95% CI = 0.24-0.87, *P = *0.017). *ATG10* rs1864182 was significantly associated with 2.27-fold elevated risk of primary gefitinib resistance (95% CI = 1.04-4.97, *P = *0.040). On the contrary, *ATG10* rs1864182 might be a protective SNP for acquired gefitinib resistance (HR = 0.30, 95% CI = 0.09-0.97, *P = *0.044). Among patients with *EGFR* mutations, *ATG5* rs510432 and rs688810 genetic variations were significantly associated with primary gefitinib resistance (rs510432 A allele: HR = 0.39, 95% CI = 0.18-0.85, *P = *0.018; rs688810 C allele: HR = 3.01, 95% CI = 1.26-7.22, *P = *0.014). Additionally, *ATG10* rs10036653 was significantly associated with acquired gefitinib resistance of *EGFR* mutant patients (T allele: HR = 0.37, 95% CI = 0.19-0.72, *P = *0.004), which was verified in log-rank test (AT and TT vs. AA: 16 months vs. 6.5 months, *P* = 0.009) (Fig. 3). However, other autophagy core gene SNPs did not significantly affect primary or acquired gefitinib-resistance (all *P* > 0.05) (Supplementary Table 6 and Supplementary Table 7).

**Table 2:** Association of genetic variants of autophagy core genes with primary resistance or acquired resistance of gefitinib.

Genes	SNPs	Genotypes	Patients No. (%)	Primary resistance		Acquired resistance		
				HR (95% CI)	*P*	HR (95% CI)		*P*
*ATG5*	rs510432	GG	105	Reference		Reference		
			43(40.95)					
		GA	41(39.05)	0.54 (0.25-1.15)	0.107	0.95 (0.55-1.62)		0.838
		AA	21(20.00)	0.34 (0.13-0.93)	0.035	1.28 (0.67-2.46)		0.453
		GA + AA	62(59.05)	0.45 (0.24-0.87)	0.017	1.05 (0.65-1.69)		0.848
*ATG5*	rs688810	TT	106	Reference		Reference		
			41(38.68)					
		TC	43(40.57)	1.67 (0.77-3.63)	0.194	0.99 (0.52-1.89)		0.972
		CC	22(20.75)	1.76 (0.74-4.20)	0.200	0.96 (0.42-2.17)		0.912
		TC + CC	65(61.32)	1.70 (0.85-3.41)	0.132	1.03 (0.57-1.85)		0.920
*ATG10*	rs10036653	AA	107	Reference		Reference		
			40(37.38)					
		AT	52(48.60)	0.74 (0.35-1.54)	0.418	0.61(0.32-1.18)		0.142
		TT	15(14.02)	0.66 (0.23-1.86)	0.432	0.39 (0.13-1.20)		0.101
		AT + TT	67(62.62)	0.77 (0.40-1.50)	0.446	0.59 (0.32-1.10)		0.099
*ATG10*	rs1864182	TT	113	Reference		Reference		
			96(84.96)					
		TG	16(14.16)	2.07 (0.91-4.72)	0.082	0.30 (0.09-0.97)		0.044
		GG	1(0.88)	N.C.	N.C.	N.C.		N.C.
		TG + GG	17(15.04)	2.27 (1.04-4.97)	0.040	0.30 (0.09-0.97)		0.044
Genes	SNPs	Genotypes	Patients with *EGFR* mutation No. (%)	Primary resistance of patients with *EGFR* mutations		Acquired resistance of patients with *EGFR* mutations		
				HR (95% CI)	*P*	HR (95% CI)	*P*	
*ATG5*	rs510432	GG	78	Reference		Reference		
			33(42.31)					
		GA	28(35.90)	0.56 (0.22-1.41)	0.217	0.65 (0.26-1.62)	0.353	
		AA	17(21.79)	0.24 (0.07-0.85)	0.026	0.77 (0.29-2.05)	0.605	
		GA + AA	45(57.69)	0.39 (0.18-0.85)	0.018	0.74 (0.34-1.60)	0.448	
*ATG5*	rs688810	TT	80	Reference		Reference		
			31(38.75)					
		TC	31(38.75)	2.89 (1.05-8.01)	0.041	0.94 (0.45-1.99)	0.877	
		CC	18(22.50)	3.02 (1.11-8.19)	0.030	1.21 (0.46-3.23)	0.700	
		TC + CC	49(61.25)	3.01 (1.26-7.22)	0.014	1.07 (0.54-2.10)	0.854	
*ATG10*	rs10036653	AA	80	Reference		Reference		
			28(35.00)					
		AT	43(53.75)	0.66 (0.29-1.53)	0.332	0.41 (0.21-0.83)	0.013	
		TT	9(11.25)	0.60 (0.17-2.12)	0.423	0.29 (0.06-1.38)	0.120	
		AT + TT	52(65.00)	0.66 (0.31-1.40)	0.278	0.37 (0.19-0.72)	0.004	
*ATG10*	rs1864182	TT	81	Reference		Reference		
			70(86.42)					
		TG	10(12.35)	1.75 (0.63-4.87)	0.283	0.55 (0.17-1.81)	0.323	
		GG	1(1.23)	N.C.	N.C.	N.C.	N.C.	
		TG + GG	11(13.58)	2.00 (0.78-5.15)	0.149	0.55 (0.17-1.81)	0.323	

Hazard ratios (HRs) and 95% confidence intervals (CIs) for the association between SNPs and geifitinib-resistance were estimated by Cox regression adjusted by sex, age, smoking status, ECGO and stages. N.C., not calculated.

### ATG5 rs510432 and ATG10 rs10036653 may influence binding of transcription factors

**Figure 4:**
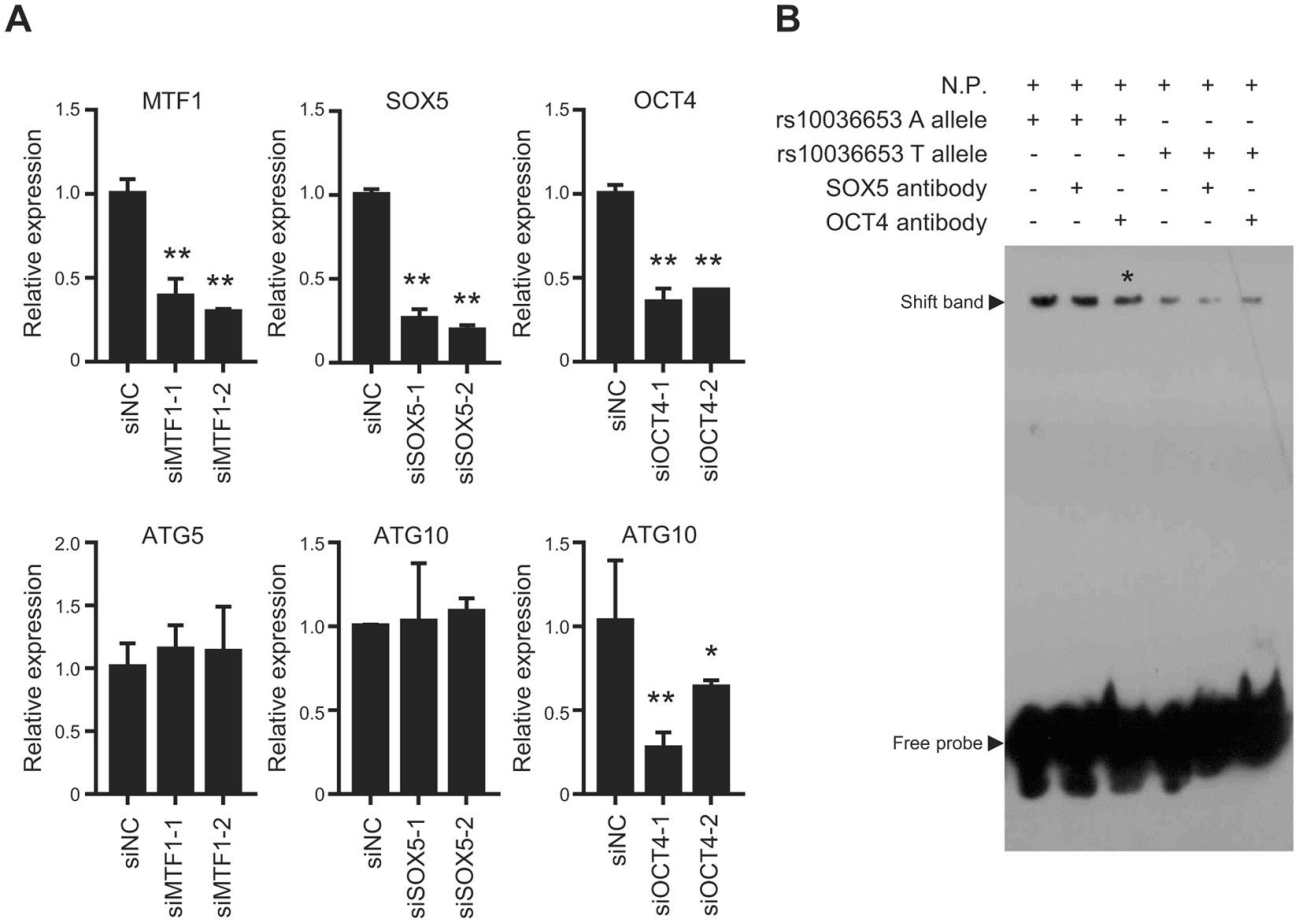
Functional evaluation of *ATG5* rs510432 and *ATG10* rs10036653 in lung cancer cells. (**A**) Relative gene expression was examined through qRT-PCR. (**B**) Electrophoretic mobility-shift assay (EMSA) with biotin-labeled rs10036653T or rs10036653A probes and A549 nuclear extracts (N.P.).

All autophagy core gene SNPs investigated in the current study were selected based on their MAF in Chinese Han population and potential function to their host genes. We found that *ATG5* rs510532 and *ATG10* rs10036653 contributed to not only survival but also drug resistance among gefitinib-treated advanced lung adenocarcinoma patients. Interestingly, both SNPs are upstream gene variants (Supplementary Table 3), which leads us to examine whether they could change transcription factor (TF) binding affinities to genomic sequences and, thus, affect gene regulation. By using RegulomeDB, an online bioinformatics tool (http://regulome.stanford.edu/)^29^, we found that *ATG5* rs510432 might change the binding ability of Mtf1 to *ATG5* promoter, and *ATG10* rs10036653 may affect the binding affinities of Sox5 as well as Oct4 to *ATG10* promoter (Table 3). We validated the bioinformatics prediction in A549 cells. After silencing endogenous expression of *OCT4*, *MTF1* or *SOX5* with siRNAs (siSOX5-1, siSOX5-2, siMTF1-1, siMTF1-2, siOCT4-1 and siOCT4-2), we found that only decreased expression of *OCT4* can suppress *ATG10* expression (Fig. 4A). These results indicate that OCT4 might acts as important TF impacting *ATG10* expression. Because *ATG10* rs10036653 SNP is located in a predicted OCT4 binding sequence, we then conducted EMSA to distinguish the differences in binding capacity between the rs10036653T or rs10036653A alleles. As shown in Fig. 4B, we found that OCT4-containing A549 nuclear extracts bound more to the biotin-labeled oligonucleotide probe with the A allele sequence compared to the T allele probe. Interestingly, although we did not find super-shift bands, we did observe attenuated OCT4 binding band with OCT4 antibody used (Fig. 4B). These observations may explain the possible correlations between these SNPs with prognosis of gefitinib treatment.

**Table 3:** Transcription factor binding site analyses of *ATG5* rs510532 and *ATG10* rs10036653.

SNPs	Score RegulomeDB*	Method	Location	Motif	Reference
*ATG5* rs510432	3a	PWM	chr6:106774020..106774034	Mtf1	^38^
*ATG10* rs10036653	6	PWM	chr5:81266375..81266390	Sox5	^39^
		PWM	chr5:81266368..81266383	Oct-4 (POU5F1)	^38^
		Footprinting	chr5:81266368..81266383	Oct-4 (POU5F1)	^40^

Note: PWM, Position-Weight Matrix for TF binding; Footprinting, DNase Footprinting.

All results were from RegulomeDB.

*“3a” means “TF binding + any motif + DNase peak” is supportive for transcription factor binding, while “6” means the results is verified by “other” methods.

## Discussions

EGFR-TKIs have been proved to be promising treatment of NSCLC, especially for lung adenocarcinoma patients harboring *EGFR* mutations. In addition to the *EGFR* mutations, we and others also found that germline variations might be prognostic markers of gefitinib treatment^22,23^. In this study, we systematically evaluated 23 SNPs from eleven autophagy core genes and treatment outcomes of advanced lung adenocarcinomas patients. Multiple genetic variations in autophagy core genes, i.e. *ATG5* rs510532 and *ATG10* rs10036653, were found to be significantly associated with clinical outcomes, especially in those with *EGFR* mutations. To the best of our knowledge, our study is the first to examine clinical implications of autophagy SNPs in patients with *EGFR* mutant adenocarcinoma.

Genetic variations of autophagy core genes are investigated in several human cancers. Qin *et al*. examined 14 potentially functional polymorphisms in six autophagy-related genes (*ATG3*, *ATG5*, *ATG7*, *ATG10*, *ATG12* and *LC3*) in breast cancer susceptibility and found that *ATG10* rs1864182 and rs10514231 were associated with significantly decreased risk of breast cancer^24^. After genotyping 40 tagging SNPs from 7 core autophagy pathway genes in 458 localized prostate cancer patients, Huang *et al*. observed the association between *ATG16L1* rs78835907 and recurrence of localized disease, which was replicated in more advanced disease^30^. White *et al*. examined five SNPs in three *ATG* genes (*ATG5*, *ATG10* and *ATG16L*) and found that *ATG* SNPs might be differentially associated with specific host and melanoma characteristics including age at diagnosis, tumor infiltrating lymphocytes, and stage^31^. Berger *et al*. genotyped 12 SNPs in eight autophagy-related genes among patients with mCRC treated with first-line FOLFIRI and bevacizumab in two phase III randomized trials and found that the *FIP200* rs1129660 variant showed significant associations with hypertension^32^. In head and neck squamous cell carcinoma, Fernández-Mateos *et al*. observed the associations between *ATG10* rs1864183 and a higher susceptibility to develop laryngeal cancer, *ATG2B* rs3759601 and pharyngeal cancer as well as *ATG16L1* rs2241880 and oral carcinoma^33^. However, it is still unclear if genetic variations of autophagy core genes would impact prognosis of advanced lung adenocarcinomas patients.

Accumulated evidences demonstrated that autophagy plays an essential role in escaping from the anti-neoplastic effects of drugs^34–36^. In NSCLC cells, gefitinib treatment can induce elevated *ATG5* expression and increased autophagy^34^. Cytotoxicity induced by gefitinib was greatly enhanced after autophagy inhibition by *ATG5* silencing^34^, which suggests that *ATG5*-regulated autophagy inhibition represents a promising approach to improve the efficacy of *EGFR*-TKIs. Similarly, Sakuma *et al*. found that depletion of *ATG5*, an autophagy inhibitor, markedly reduces gefitinib-resistant cell viability of *EGFR*-mutated lung adenocarcinoma cells under hypoxic conditions^36^. These results elucidated that *ATG5* might be a crucial gene impacting clinical outcomes of gefitinib treatments. As a result, it is biologically plausible that the potential functional *ATG5* rs510532 genetic variant may also be a prognostic marker for gefitinib therapy.

*ATG10* is an E2-like enzyme involved in E2 ubiquitin-like modifications essential for autophagosome formation. Jo *et al*. found that ATG10 was increased in colorectal cancer and associated with lymphovascular invasion and lymph node metastasis^37^. Qin *et al*. demonstrated that potentially functional polymorphisms in *ATG10* were associated with risk of breast cancer in a Chinese population^24^. These results indicated that *ATG10* and its genetic polymorphisms might be an important component during carcinogenesis. In line with this, we observed significant association between the *ATG10* rs1864182 SNP with prolonged survival and gefitinib-resistance of *EGFR* mutant NSCLC patients.

In summary, *ATG5* rs510532 and *ATG10* rs10036653 genetic variations in autophagy core genes are significantly associated with clinical outcomes of advanced lung adenocarcinoma treated with gefitinib. Genotyping of these genetic variations with detection of *EGFR* mutations may improve the prediction of the treatment outcomes. Our study also highlights the possibility of patient-tailored decisions especially during EGFR-TKIs based on combination of germline and somatic variation detection.

## Electronic supplementary material

Supplementary information
