# Supplementary material for: Clinical Implications of the Autophagy Core Gene Variations in Advanced Lung Adenocarcinoma Treated with Gefitinib
Source: Sci Rep. 2017 Dec 19;7:17814. doi: 10.1038/s41598-017-18165-5 (PMC5736620; doi:10.1038/s41598-017-18165-5)

Clinical Implications of the Autophagy Core Gene Variations in Advanced Lung Adenocarcinoma Treated with Gefitinib

Jupeng Yuan[1,†]; Nasha Zhang [1,2,†]; Longbin Yin [3,†]; Hui Zhu[2]; Li Zhang[3]; Liqing Zhou[4]; Ming Yang[1,*]

Authors' affiliations: [1]Shandong Provincial Key Laboratory of Radiation Oncology, Cancer Research Center, Shandong Cancer Hospital affiliated to Shandong University, Shandong Academy of Medical Sciences, Jinan, Shandong Province, China; [2]Department of Radiation Oncology, Shandong Cancer Hospital affiliated to Shandong University, Shandong Academy of Medical Sciences, Jinan, Shandong Province, China; [3]Department of Oncology, Tongji Hospital, Tongji Medical College, Huazhong University of Science and Technology, Wuhan, Hubei Province, China; [4]Department of Radiation Oncology, Huaian No. 2 Hospital, Huaian, Jiangsu Province, China.

[†]**Note:** Jupeng Yuan, Nasha Zhang and Longbin Yin contributed equally to this work.

[*]**Correspondence to:** Ming Yang, Professor, Shandong Provincial Key Laboratory of Radiation Oncology, Cancer Research Center, Shandong Cancer Hospital affiliated to Shandong University, Shandong Academy of Medical Sciences, Jinan 250117, Shandong Province, China. Tel & Fax: 86531-67626536; E-mail: aaryoung@yeah.net, yangm@sdu.edu.cn.

Short title: ATG5 and ATG10 in EGFR-TKI therapy

Keywords: EGFR-TKI; gefitinib; autophagy; genetic polymorphism; survival; drug resistance

Manuscript category: Research Article

Conflict of Interest: None to declare

Funding: This study was partially supported by National Natural Science Foundation of China (31671300, 81572934); Taishan Scholars Program of Shandong Province (tsqn20161060); the National High-Tech Research and Development Program of China (2015AA020950); the Science and Technology Planning Project of Huaian (HAS2015013); Foundation of Shandong Academy of Medical Sciences (2015-44); Medicine and Health Science Technology Foundation (2015WSA18054).

Supplementary Table 1. Clinical characteristics of advanced lung adenocarcinoma patients treated with gefitinib

**Characteristics**	***n* (%)**
	108
Age of onset (year)	
Average	56
Range	46-66
Gender	
Male	53(49.07)
Female	55(50.93)
Treatment with gefitinib	
Yes	108(100.00)
No	0(0.00)
ECOG	
0-1	86(79.63)
≥ 2	22(20.37)
Smoke	
Yes	32(29.63)
No	76(70.37)
EGFR status	
WT	27(25.00)
Mutant	81(75.00)
EGFR mutation	
Del 19	34(31.48)
L858R	37(34.26)
Others	10(9.26)
Histological grade (TNM)	
IIIB	25(23.15)
IV	83(76.85)
Treatment effects	
CR	9(8.33)
PR	28(25.93)
SD	34(31.48)
PD	37(34.26)

Note: EGFR, epidermal growth factor receptor; TNM, tumor node metastasis; CR, complete response; PR, partial response; SD, stable disease; PD, progressive disease.

Supplementary Table 2. Oligonucleotides used in this study

#	Direction	Sequence
qRT-PCR		
OCT4	Forward	GGTTCTATTTGGGAAGGTATTCAG
	Reverse	TGGTTCGCTTTCTCTTTCGG
MTF1	Forward	GCAGCAAATACTTCACCACAC
	Reverse	GAGTGATTTGTATCCTCTGATCC
SOX5	Forward	CTGGATGTAGTGACCCTTACC
	Reverse	CTGTTTGTGCTCTTGTCTGTG
ATG5	Forward	GTTGCTTACTAAGTTTGGCTTTGG
	Reverse	GGTGTGCCTTCATATTCAAACC
ATG10	Forward	TATTACGCAACAGGAACATCCA
	Reverse	GAGTCCGCTTTAAGGTTGTC
β-actin	Forward	GGCGGCACCACCATGTACCCT
	Reverse	AGGGGCCGGACTCGTCATACT
EMSA		
rs10036653-A	Forward	GTTTTTGGCATGTGAATTATGAAACTTTAAA
	Reverse	TTTAAAGTTTCATAATTCACATGCCAAAAAC
rs10036653-T	Forward	GTTTTTGGCATGTGATTTATGAAACTTTAAA
	Reverse	TTTAAAGTTTCATAAATCACATGCCAAAAAC
siRNA duplexes		
siSOX5-1	Sense	GCCAUAUUAUGAGGAGCAATT
	Antisense	UUGCUCCUCAUAAUAUGGCTT
siSOX5-2	Sense	CCUGUUAUCCAGAGCACUUTT
	Antisense	AAGUGCUCUGGAUAACAGGTT
siMTF1-1	Sense	CCAGCAAUCAUCUUUGAAUTT
	Antisense	AUUCAAAGAUGAUUGCUGGTT
siMTF1-2	Sense	GCACACAGUUUGCUGCUAATT
	Antisense	UUAGCAGCAAACUGUGUGCTT
siOCT4-1	Sense	GAGGCAACCUGGAGAAUUUTT
	Antisense	AAAUUCUCCAGGUUGCCUCTT
siOCT4-2	Sense	GCUCUCCCAUGCAUUCAAATT
	Antisense	UUUGAAUGCAUGGGAGAGCTT

Supplementary Table 3. Autophagy core gene SNPs analyzed in this study

No	Genes	SNPs	Variant type	Alleles	Location
1	*ATG2B*	rs3759601	Missense variant	G/C	Chr14:96311131
2	*ATG2B*	rs9323945	Missense variant	T/C	Chr14:96315575
3	*ATG3*	rs7652377	Intron variant	A/C	Chr3:112564045
4	*ATG4C*	rs10493327	Intron variant	C/T	Chr1:62838174
5	*ATG5*	rs2245214	Intron variant	C/G	Chr6:106214866
6	*ATG5*	rs3804338	Intron variant	C/T	Chr6:106324898
7	*ATG5*	rs510432	Upstream gene variant	T/C	Chr6:106326155
8	*ATG5*	rs688810	Upstream gene variant	A/G	Chr6:106328206
9	*ATG7*	rs1375206	Intron variant	C/G	Chr3:11297643
10	*ATG7*	rs1470612	Intron variant	C/T	Chr3:11295492
11	*ATG7*	rs8154	Synonymous variant	T/C	Chr3:11554828
12	*ATG9B*	rs61733329	Missense variant	C/T	Chr7:151017080
13	*ATG10*	rs10036653	Upstream gene variant	A/T	Chr5:81970563
14	*ATG10*	rs10514231	Intron variant	C/T	Chr5:82011593
15	*ATG10*	rs1864182	Missense variant	C/A	Chr5:82253421
16	*ATG10*	rs1864183	Missense variant	C/T	Chr5:82253397
17	*ATG10*	rs3734114	Missense variant	T/C	Chr5:82058570
18	*ATG12*	rs26532	Noncoding transcript exon variant	C/A	Chr5:115837981
19	*ATG12*	rs26534	Intron variant	G/A	Chr5:115840004
20	*ATG12*	rs26538	Intron variant	C/T	Chr5:115842698
21	*ATG16L1*	rs2241880	Missense variant	A/G	Chr2:233274722
22	*ATG16L2*	rs11235604	Missense variant	C/T	Chr11:72822491
23	*BECN*	rs9890617*	Noncoding transcript exon variant	C/T	Chr17:42820651
24	*BECN*	rs9891429*	Noncoding transcript exon variant	T/C	Chr17:42820407
25	*BECN*	rs10512488*	Noncoding transcript exon variant	G/A	Chr17:42811886
26	*BECN*	rs11552192	3' UTR variant	A/T	Chr17:42810491

Note: SNP, single nucleotide polymorphism; Chr, Chromosome.
**BECN* rs9890617, rs9891429 and rs10512488 were excluded for the following analyses because of genotyping failure.

Supplementary Table 4. Associations of genetic variants of autophagy core genes with OS and PFS

Genes	SNPs	Genotypes	Patients	OS of all patients		PFS of all patients	
			No. (%)	HR (95% CI)	*P*	HR (95% CI)	*P*
				107			
		CC	45(42.06)	Reference		Reference	
ATG2B	rs3759601	CG	8(7.48)	1.09 (0.71-1.67)	0.705	1.08 (0.69-1.68)	0.742
		GG	54(50.47)	1.69 (0.73-3.88)	0.218	1.84 (0.75-4.52)	0.181
		CG+GG	62(57.94)	1.13 (0.75-1.70)	0.561	1.13 (0.74-1.73)	0.580
				108			
		CC	65(60.19)	Reference		Reference	
ATG2B	rs9323945	CT	38(35.19)	0.75 (0.46-1.21)	0.239	0.71 (0.43-1.16)	0.170
		TT	5(4.63)	0.66 (0.25-1.71)	0.393	0.47 (0.16-1.35)	0.161
		CT+TT	43(39.81)	0.74 (0.47-1.16)	0.186	0.69 (0.43-1.09)	0.111
				107			
		CC	54(50.47)	Reference		Reference	
ATG3	rs7652377	CA	46(42.99)	0.84 (0.54-1.29)	0.416	0.88 (0.56-1.38)	0.580
		AA	7(6.54)	0.50 (0.21-1.22)	0.129	0.45 (0.17-1.19)	0.107
		CA+AA	53(49.53)	0.78 (0.51-1.18)	0.234	0.81 (0.63-1.25)	0.337
				104			
		AA	53(50.96)	Reference		Reference	
ATG4C	rs10493327	AG	35(33.65)	1.21 (1.21-0.73)	0.466	1.37 (0.79-2.37)	0.263
		GG	17(16.35)	0.91 (0.49-1.66)	0.748	1.18 (0.61-2.27)	0.621
		AG+GG	52(50.00)	1.01 (0.66-1.78)	0.971	1.25 (0.77-2.02)	0.365
				106			
		GG	33(31.13)	Reference		Reference	
ATG5	rs2245214	GC	40(37.74)	0.61 (0.36-1.06)	0.078	0.73 (0.42-1.27)	0.261
		CC	33(31.13)	0.77 (0.44-1.32)	0.338	0.81 (0.46-1.44)	0.476
		GC+CC	73(68.87)	0.73 (0.46-1.15)	0.173	0.81 (0.50-1.32)	0.398
				107			
ATG5	rs3804338	CC	84(78.50)	Reference		Reference	
		CT	20(18.69)	1.26 (0.76-2.11)	0.375	1.25 (0.73-2.14)	0.418

		TT	3(2.80)	N.C.	N.C.	N.C.	N.C.
		CT+TT	23(21.50)	1.02 (0.63-1.67)	0.931	0.97 (0.58-1.65)	0.918
				104			
		CC	36(34.62)	Reference		Reference	
ATG7	rs1375206	CG	56(53.85)	0.80 (0.51-1.25)	0.321	0.35 (0.50-1.27)	0.799
		GG	12(11.54)	1.39 (0.65-2.95)	0.397	1.60 (0.73-3.50)	0.74
		CG+GG	68(65.38)	0.87 (0.57-1.35)	0.539	0.89 (0.57-1.40)	0.620
				107			
		GG	45(42.06)	Reference		Reference	
ATG7	rs1470612	GA	53(49.53)	0.82 (0.53-1.26)	0.359	0.79 (0.50-1.23)	0.291
		AA	9(8.41)	1.05 (0.46-2.39)	0.902	1.23 (0.53-2.83)	0.630
		GA+AA	62(57.94)	0.86 (0.56-1.30)	0.462	0.85 (0.55-1.31)	0.454
				107			
		CC	91(85.05)	Reference		Reference	
ATG9B	rs61733329	CT	15(14.02)	1.17 (0.64-2.14)	0.610	1.34 (0.71-2.52)	0.367
		TT	1(0.93)	N.C.	N.C.	N.C.	N.C.
		CT+TT	16(14.95)	1.23 (0.68-2.21)	0.492	1.40 (0.76-2.60)	0.284
				107			
		TT	92(85.98)	Reference		Reference	
ATG10	rs10514231	TC	13(12.15)	1.09 (1.14-3.31)	0.799	1.07 (0.54-2.13)	0.844
		CC	2(1.87)	N.C.	N.C.	N.C.	N.C.
		TC+CC	15(14.02)	0.84 (0.45-1.56)	0.578	0.98 (0.51-1.86)	0.939
				108			
		TT	91(84.26)	Reference		Reference	
ATG10	rs1864182	TG	16(14.81)	0.75 (0.40-1.40)	0.361	0.79 (0.41-1.55)	0.495
		GG	1(0.93)	N.C.	N.C.	N.C.	N.C.
		TG+GG	17(15.74)	0.82 (0.45-1.50)	0.515	0.87 (0.46-1.66)	0.675
				106			
		AA	80(75.47)	Reference		Reference	
ATG10	rs1864183	AG	25(23.58)	0.90 (0.55-1.47)	0.672	0.88 (0.52-1.50)	0.646
		GG	1(0.94)	N.C.	N.C.	N.C.	N.C.
		AG+GG	26(24.53)	0.94 (0.58-1.53)	0.797	0.92 (0.55-1.55)	0.765

				106			
		TT	69(65.09)	Reference		Reference	
ATG10	rs3734114	TC	33(31.13)	0.94 (0.58-1.50)	0.778	1.10 (0.67-1.80)	0.711
		CC	4(3.77)	N.C.	N.C.	N.C.	N.C.
		TC+CC	27(34.91)	0.96 (0.61-1.51)	0.857	1.13 (0.70-1.81)	0.624
				107			
		AA	46(42.99)	Reference		Reference	
ATG12	rs26532	AC	42(39.25)	1.07 (0.67-1.71)	0.788	1.02 (0.61-1.71)	0.932
		CC	19(17.76)	1.16 (0.63-2.13)	0.645	1.18 (0.63-2.21)	0.606
		AC+CC	61(57.01)	1.11 (0.72-1.71)	0.643	1.05 (0.66-1.66)	0.837
				103			
		GG	38(36.89)	Reference		Reference	
ATG12	rs26534	GA	50(48.54)	1.09 (0.68-1.73)	0.727	1.09 (0.68-1.75)	0.729
		AA	15(14.56)	1.27 (0.64-2.54)	0.496	1.17 (0.59-2.32)	0.664
		GA+AA	65(63.11)	1.03 (0.68-1.58)	0.876	1.03 (0.67-1.58)	0.911
				106			
		AA	68(64.15)	Reference		Reference	
BECN	rs11552192	AT	34(32.08)	1.01 (0.65-1.57)	0.965	0.83 (0.52-1.33)	0.444
		TT	4(3.77)	N.C.	N.C.	N.C.	N.C.
		AT+TT	38(35.85)	1.10 (0.72-1.67)	0.669	0.94 (0.61-1.47)	0.796

Note: SNP, single nucleotide polymorphism; PFS, progression–free survival time; OS, overall survival time; HR, hazard ratio; CI, confidence interval; N.C., not calculated.

Hazard ratios (HRs) and 95% confidence intervals (CIs) for the association between SNPs and PFS as well as OS were estimated by Cox regression adjusted by sex, age, smoking status, ECGO and stages.

Supplementary Table 5. Associations of genetic variants of autophagy core genes with OS and PFS in patients with *EGFR* mutations

Genes	SNPs	Genotypes	Patients No. (%)	OS of patients with EGFR mutation		PFS of patients with EGFR mutation	
				HR (95% CI)	*P*	HR (95% CI)	*P*
				80			
ATG2B	rs3759601	CC	40(50.00)	Reference		Reference	
		CG	34(42.50)	1.23 (0.75-2.03)	0.413	1.12 (0.68-1.85)	0.665
		GG	6(7.50)	1.25 (0.47-3.30)	0.653	1.25 (0.44-4.55)	0.675
		CG+GG	40(50.)	1.25 (0.77-2.01)	0.369	1.13 (0.70-1.83)	0.624
				81			
ATG2B	rs9323945	CC	50(61.73)	Reference		Reference	
		CT	27(33.33)	1.13 (0.65-1.97)	0.658	1.19 (0.69-2.06)	0.526
		TT	4(4.94)	N.C.	N.C.	N.C.	N.C.
		CT+TT	31(38.27)	1.07 (0.64-1.77)	0.805	1.12 (0.86-1.86)	0.660
				80			
ATG3	rs7652377	CC	37(46.25)	Reference		Reference	
		CA	37(46.25)	0.71 (0.42-1.19)	0.189	0.73 (0.42-1.25)	0.251
		AA	6(7.50)	0.48 (0.18-1.29)	0.146	0.58 (0.21-1.59)	0.286
		CA+AA	43(53.75)	0.68 (0.41-1.12)	0.129	0.73 (0.43-1.23)	0.230
				78			
ATG4C	rs10493327	AA	42(53.85)	Reference		Reference	
		AG	24(30.77)	1.24 (0.69-2.24)	0.472	1.47 (0.79-2.77)	0.226
		GG	12(15.38)	0.78 (0.37-1.65)	0.516	0.86 (0.40-1.88)	0.710
		AG+GG	36(46.15)	1.04 (0.61-1.77)	0.882	1.20 (0.68-2.12)	0.519
				80			
ATG5	rs2245214	GG	26(32.50)	Reference		Reference	
		GC	29(36.25)	0.59 (0.32-1.10)	0.095	0.60 (0.32-1.12)	0.110
		CC	25(31.25)	0.66 (0.35-1.24)	0.196	0.59 (0.31-1.13)	0.113
		GC+CC	54(67.50)	0.62 (0.37-1.03)	0.066	**0.57 (0.34-0.97)**	**0.037**
ATG5	rs3804338			80			
		CC	64(80.00)	Reference		Reference	

		CT	14(17.50)	1.21 (0.66-2.22)	0.529	1.38 (0.75-2.55)	0.304
		TT	2(2.50)	N.C.	N.C.	N.C.	N.C.
		CT+TT	16(20.00)	0.95 (0.54-1.70)	0.873	1.10 (0.60-2.02)	0.750
				78			
		CC	27(34.62)	Reference		Reference	
ATG7	rs1375206	CG	41(52.56)	0.74 (0.43-1.25)	0.259	0.82 (0.48-1.41)	0.473
		GG	10(12.82)	1.31 (0.56-3.04)	0.533	1.50 (0.63-3.61)	0.361
		CG+GG	51(65.38)	0.82 (0.50-1.37)	0.449	0.92 (0.55-1.54)	0.756
				80			
		GG	35(43.75)	Reference		Reference	
ATG7	rs1470612	GA	38(47.50)	0.76 (0.45-1.28)	0.302	0.79 (0.47-1.32)	0.366
		AA	7(8.75)	0.90 (0.35-2.30)	0.831	1.08 (0.12-2.78)	0.874
		GA+AA	45(56.25)	0.80 (0.49-1.32)	0.386	0.85 (0.52-1.40)	0.523
				80			
		CC	67(83.75)	Reference		Reference	
ATG9B	rs61733329	CT	12(15.00)	1.33 (0.68-2.61)	0.405	1.72 (0.87-3.40)	0.121
		TT	1(1.25)	N.C.	N.C.	N.C.	N.C.
		CT+TT	13(16.25)	1.41 (0.73-2.70)	0.305	1.79 (0.92-3.49)	0.085
				80			
		TT	70(87.50)	Reference		Reference	
ATG10	rs10514231	TC	9(11.25)	1.01 (0.46-2.24)	0.980	0.92 (0.41-2.07)	0.841
		CC	1(1.25)	N.C.	N.C.	N.C.	N.C.
		TC+CC	10(12.50)	1.14 (0.54-2.43)	0.726	1.05 (0.49-2.25)	0.909
				81			
		TT	70(86.42)	Reference		Reference	
ATG10	rs1864182	TG	10(12.35)	1.13 (0.54-2.40)	0.742	1.03 (0.48-2.20)	0.949
		GG	1(1.23)	N.C.	N.C.	N.C.	N.C.
		TG+GG	11(13.58)	1.26 (0.62-2.58)	0.526	1.14 (0.55-2.36)	0.720
				79			
		AA	60(75.79)	Reference		Reference	
ATG10	rs1864183	AG	18(22.78)	1.30 (0.73-2.33)	0.373	1.20 (0.67-2.14)	0.550
		GG	1(1.27)	N.C.	N.C.	N.C.	N.C.

		AG+GG	19(24.05)	1.36 (0.77-2.41)	0.287	1.25 (0.71-2.21)	0.446
				79			
		TT	54(68.35)	Reference		Reference	
ATG10	rs3734114	TC	22(27.85)	0.85 (0.48-1.53)	0.594	0.90 (0.50-1.62)	0.726
		CC	3(3.80)	N.C.	N.C.	N.C.	N.C.
		TC+CC	25(31.65)	0.92 (0.54-1.60)	0.777	0.94 (0.55-1.63)	0.836
				81			
		AA	35(43.21)	Reference		Reference	
ATG12	rs26532	AC	31(38.27)	1.03 (0.60-1.77)	0.922	1.01 (0.56-1.82)	0.986
		CC	15(18.52)	1.07 (0.53-2.13)	0.859	1.07 (0.53-2.15)	0.852
		AC+CC	46(56.79)	1.05 (0.64-1.72)	0.853	0.98 (0.59-1.65)	0.951
				77			
		GG	28(36.36)	Reference		Reference	
ATG12	rs26534	GA	38(49.35)	1.01 (0.59-1.73)	0.975	1.15 (0.67-2.00)	0.611
		AA	11(14.29)	0.92 (0.39-2.19)	0.851	0.91 (0.40-2.10)	0.831
		GA+AA	49(63.64)	0.95 (0.58-1.55)	0.823	1.02 (0.62-1.68)	0.942
				80			
		AA	50(62.50)	Reference		Reference	
BECN	rs11552192	AT	28(35.00)	1.11 (0.66-1.88)	0.693	0.98 (0.57-1.67)	0.937
		TT	2(2.50)	N.C.	N.C.	N.C.	N.C.
		AT+TT	30(37.50)	1.19 (0.71-1.98)	0.519	1.06 (0.63-1.78)	0.827

Note: SNP, single nucleotide polymorphism; PFS, progression–free survival time; OS, overall survival time; HR, hazard ratio; CI, confidence interval; N.C., not calculated.

Hazard ratios (HRs) and 95% confidence intervals (CIs) for the association between SNPs and PFS as well as OS were estimated by Cox regression adjusted by sex, age, smoking status, ECGO and stages.

Supplementary Table 6. Associations of genetic variants of autophagy core genes with primary resistance and acquired resistance of gefitinib in all patients

Genes	SNPs	Genotypes	Patients No. (%)	Primary resistance of all patients		Acquired resistance of all patients	
				HR (95% CI)	*P*	HR (95% CI)	*P*
				107			
		CC	45(42.06)	Reference		Reference	
ATG2B	rs3759601	CG	8(7.48)	1.00 (0.50-2.00)	0.992	1.23 (0.69-2.21)	0.486
		GG	54(50.47)	2.09 (0.60-7.24)	0.245	1.88 (0.50-7.03)	0.348
		CG+GG	62(57.94)	1.37 (0.67-2.76)	0.718	1.24 (0.70-2.20)	0.453
				108			
		CC	65(60.19)	Reference		Reference	
ATG2B	rs9323945	CT	38(35.19)	1.04 (0.52-2.07)	0.923	0.55 (0.26-1.15)	0.110
		TT	5(4.63)	N.C.	N.C.	0.57 (0.19-1.74)	0.321
		CT+TT	43(39.81)	0.93 (0.46-1.85)	0.825	0.61 (0.32-1.17)	0.136
				107			
		CC	54(50.47)	Reference		Reference	
ATG3	rs7652377	CA	46(42.99)	1.06 (0.54-2.10)	0.868	0.88 (0.48-1.60)	0.676
		AA	7(6.54)	0.22 (0.03-1.75)	0.151	0.97 (0.31-3.06)	0.953
		CA+AA	53(49.53)	0.87 (0.45-1.68)	0.672	0.67 (0.48-1.55)	0.630
				104			
		AA	53(50.96)	Reference		Reference	
ATG4C	rs10493327	AG	35(33.65)	1.10 (0.46-2.61)	0.830	1.70 (0.81-3.57)	0.164
		GG	17(16.35)	1.62 (0.69-3.79)	0.266	0.75 (0.24-2.39)	0.628
		AG+GG	52(50.00)	1.43 (0.68-2.97)	0.345	1.26 (0.65-2.45)	0.493
				106			
		GG	33(31.13)	Reference		Reference	
ATG5	rs2245214	GC	40(37.74)	0.53 (0.24-1.19)	0.124	0.84 (0.37-1.90)	0.670
		CC	33(31.13)	**0.32 (0.13-0.79)**	**0.013**	1.46 (0.65-3.30)	0.365
		GC+CC	73(68.87)	**0.44 (0.22-0.89)**	**0.022**	1.22 (0.61-2.45)	0.583
ATG5	rs3804338			107			

		CC	84(78.50)	Reference		Reference	
		CT	20(18.69)	1.52 (0.66-3.51)	0.326	1.32 (0.64-2.71)	0.456
		TT	3(2.80)	N.C.	N.C.	N.C.	N.C.
		CT+TT	23(21.50)	1.24 (0.55-2.83)	0.605	0.94 (0.47-1.90)	0.868
				104			
		CC	36(34.62)	Reference		Reference	
ATG7	rs1375206	CG	56(53.85)	0.78 (0.36-1.69)	0.529	0.74 (0.40-1.35)	0.326
		GG	12(11.54)	1.13 (0.37-3.45)	0.834	1.94 (0.61-6.13)	0.261
		CG+GG	68(65.38)	0.84 (0.41-1.75)	0.647	0.83 (0.46-1.49)	0.534
				107			
		GG	45(42.06)	Reference		Reference	
ATG7	rs1470612	GA	53(49.53)	1.01 (0.51-2.03)	0.968	0.65 (0.35-1.18)	0.156
		AA	9(8.41)	0.78 (0.21-2.84)	0.701	1.78 (0.59-5.39)	0.305
		GA+AA	62(57.94)	1.00 (0.51-1.97)	0.991	0.73 (0.41-1.30)	0.284
				108			
		TT	69(63.89)	Reference		Reference	
ATG7	rs8154	TC	37(34.26)	0.68 (0.33-1.41)	0.298	0.78 (0.43-1.42)	0.416
		CC	2(1.85)	N.C.	N.C.	N.C.	N.C.
		TC+CC	39(36.11)	0.70 (0.35-1.40)	0.309	0.81 (0.46-1.44)	0.473
				107			
		CC	91(85.05)	Reference		Reference	
ATG9B	rs61733329	CT	15(14.02)	1.87 (0.71-4.93)	0.205	0.95 (0.42-2.17)	0.903
		TT	1(0.93)	N.C.	N.C.	N.C.	N.C.
		CT+TT	16(14.95)	2.05 (0.81-5.16)	0.128	0.95 (0.42-2.17)	0.903
				107			
		TT	92(85.98)	Reference		Reference	
ATG10	rs10514231	TC	13(12.15)	2.38 (0.98-5.78)	0.057	0.54 (0.17-1.69)	0.289
		CC	2(1.87)	N.C.	N.C.	N.C.	N.C.
		TC+CC	15(14.02)	2.19 (0.96-5.01)	0.063	0.47 (0.15-1.42)	0.179
				106			
ATG10	rs1864183	AA	80(75.47)	Reference		Reference	
		AG	25(23.58)	1.34 (0.62-2.90)	0.465	0.74 (0.35-1.59)	0.440

		GG	1(0.94)	N.C.	N.C.	N.C.	N.C.
		AG+GG	26(24.53)	1.44 (0.68-3.04)	0.343	0.74 (0.35-1.59)	0.440
				106			
		TT	69(65.09)	Reference		Reference	
ATG10	rs3734114	TC	33(31.13)	1.46 (0.71-3.01)	0.310	0.93 (0.47-1.87)	0.840
		CC	4(3.77)	N.C.	N.C.	N.C.	N.C.
		TC+CC	27(34.91)	1.37 (0.67-2.76)	0.388	1.01 (0.53-1.94)	0.970
				107			
		AA	46(42.99)	Reference		Reference	
ATG12	rs26532	AC	42(39.25)	1.14 (0.52-2.49)	0.745	0.91 (0.47-1.78)	0.789
		CC	19(17.76)	0.82 (0.32-2.14)	0.689	1.79 (0.75-4.32)	0.193
		AC+CC	61(57.01)	1.09 (0.55-2.15)	0.808	1.22 (0.50-1.68)	0.787
				103			
		GG	38(36.89)	Reference		Reference	
ATG12	rs26534	GA	50(48.54)	1.25 (0.59-2.65)	0.560	0.98 (0.53-1.82)	0.947
		AA	15(14.56)	1.23 (0.49-3.09)	0.657	1.39 (0.47-4.17)	0.554
		GA+AA	65(63.11)	1.25 (0.64-2.45)	0.521	0.94 (0.53-1.67)	0.840
				107			
		CC	40(37.38)	Reference		Reference	
ATG12	rs26538	CT	54(50.47)	1.28 (0.61-2.67)	0.515	0.90 (0.49-1.65)	0.734
		TT	13(12.15)	1.98 (0.71-5.53)	0.194	3.40 (1.00-11.63)	0.051
		CT+TT	67(62.62)	1.41 (0.72-2.74)	0.319	1.03 (0.58-1.83)	0.915
				106			
		TT	44(41.51)	Reference		Reference	
ATG16L1	rs2241880	TC	44(41.51)	1.17 (0.57-2.39)	0.670	1.62 (0.83-3.17)	0.159
		CC	18(16.98)	1.35 (0.48-3.84)	0.571	1.94 (0.82-4.58)	0.131
		TC+CC	62(58.49)	1.20 (0.62-2.34)	0.593	1.50 (0.84-2.69)	0.171
				108			
		CC	89(82.41)	Reference		Reference	
ATG16L2	rs11235604	CT	18(16.67)	1.37 (0.66-2.86)	0.401	1.81 (0.88-3.70)	0.107
		TT	1(0.93)	N.C.	N.C.	N.C.	N.C.
		CT+TT	19(17.60)	1.45 (0.72-2.95)	0.301	1.81 (0.88-3.70)	0.107

				106			
		AA	68(64.15)	Reference		Reference	
BECN	rs11552192	AT	34(32.08)	1.13 (0.54-2.36)	0.744	0.70 (0.38-1.29)	0.251
		TT	4(3.77)	N.C.	N.C.	N.C.	N.C.
		AT+TT	38(35.85)	1.35 (0.68-2.65)	0.390	0.75 (0.41-1.37)	0.349

Note: SNP, single nucleotide polymorphism; HR, hazard ratio; CI, confidence interval; N.C., not calculated.

Hazard ratios (HRs) and 95% confidence intervals (CIs) for the association between SNPs and geifitinib-resistance were estimated by Cox regression adjusted by sex, age, smoking status, ECGO and stages.

Supplementary Table 7. Associations of genetic variants of autophagy core genes with primary resistance and acquired resistance of gefitinib in patients with *EGFR* mutations

Genes	SNPs	Genotypes	Patients with EGFR mutation No. (%)	Primary resistance of patients with EGFR mutation		Acquired resistance of patients with EGFR mutation	
				HR (95% CI)	***P***	HR (95% CI)	***P***
				80			
		CC	40(50.00)	Reference		Reference	
ATG2B	rs3759601	CG	34(42.50)	1.41 (0.64-3.12)	0.393	0.96 (0.49-1.85)	0.891
		GG	6(7.50)	1.11 (0.21-5.76)	0.900	1.79 (0.47-6.80)	0.395
		CG+GG	40(50.)	1.42 (0.66-3.07)	0.372	0.98 (0.52-1.86)	0.961
				81			
		CC	50(61.73)	Reference		Reference	
ATG2B	rs9323945	CT	27(33.33)	0.51 (0.70-3.25)	0.297	1.24 (0.55-2.79)	0.602
		TT	4(4.94)	N.C.	N.C.	N.C.	N.C.
		CT+TT	31(38.27)	1.31 (0.61-2.83)	0.487	1.28 (0.65-2.53)	0.484
				80			
		CC	37(46.25)	Reference		Reference	
ATG3	rs7652377	CA	37(46.25)	1.07 (0.49-2.33)	0.868	0.60 (0.29-1.25)	0.174
		AA	6(7.50)	0.29 (0.03-2.69)	0.279	1.17 (0.34-4.00)	0.803
		CA+AA	43(53.75)	0.90 (0.42-1.92)	0.777	0.68 (0.33-1.39)	0.285
				78			
		AA	42(53.85)	Reference		Reference	
ATG4C	rs10493327	AG	24(30.77)	1.48 (0.55-3.96)	0.436	1.96 (0.82-4.68)	0.130
		GG	12(15.38)	1.14 (0.39-3.37)	0.810	0.68 (0.20-2.33)	0.543
		AG+GG	36(46.15)	1.55 (0.64-3.73)	0.332	1.37 (0.62-3.02)	0.440
				80			
		GG	26(32.50)	Reference		Reference	
ATG5	rs2245214	GC	29(36.25)	0.63 (0.26-1.52)	0.306	0.44 (0.17-1.16)	0.097
		CC	25(31.25)	**0.22 (0.67-0.70)**	**0.011**	0.98 (0.40-2.42)	0.970
		GC+CC	54(67.50)	**0.43 (0.20-0.94)**	**0.035**	0.67 (0.32-1.41)	0.289

				80			
		CC	64(80.00)	Reference		Reference	
ATG5	rs3804338	CT	14(17.50)	1.19 (0.43-3.28)	0.741	1.66 (0.73-3.75)	0.226
		TT	2(2.50)	N.C.	N.C.	N.C.	N.C.
		CT+TT	16(20.00)	0.95 (0.35-2.59)	0.917	1.39 (0.63-3.09)	0.415
				78			
		CC	27(34.62)	Reference		Reference	
ATG7	rs1375206	CG	41(52.56)	0.76 (0.30-1.95)	0.566	0.83 (0.42-1.62)	0.580
		GG	10(12.82)	1.25 (0.37-4.21)	0.725	1.38 (0.34-5.67)	0.651
		CG+GG	51(65.38)	0.88 (0.37-2.12)	0.778	0.93 (0.48-1.80)	0.836
				80			
		GG	35(43.75)	Reference		Reference	
ATG7	rs1470612	GA	38(47.50)	1.09 (0.47-2.53)	0.841	0.70 (0.36-1.36)	0.286
		AA	7(8.75)	0.97 (0.25-3.76)	0.969	1.23 (0.32-4.75)	0.768
		GA+AA	45(56.25)	1.11 (0.50-2.49)	0.799	0.79 (0.42-1.49)	0.461
				81			
		TT	55(67.90)	Reference		Reference	
ATG7	rs8154	TC	26(32.50)	0.53 (0.22-1.28)	0.159	0.75 (0.37-1.53)	0.429
		CC	0(0.00)	N.C.	N.C.	N.C.	N.C.
		TC+CC	26(32.50)	0.53 (0.22-1.28)	0.159	0.75 (0.37-1.53)	0.429
				80			
		CC	67(83.75)	Reference		Reference	
ATG9B	rs61733329	CT	12(15.00)	2.13 (0.79-5.73)	0.135	1.23 (0.47-3.21)	0.680
		TT	1(1.25)	N.C.	N.C.	N.C.	N.C.
		CT+TT	13(16.25)	2.29 (0.89-5.92)	0.086	1.23 (0.47-3.21)	0.680
				80			
		TT	70(87.50)	Reference		Reference	
ATG10	rs10514231	TC	9(11.25)	1.44 (0.46-4.53)	0.529	0.57 (0.17-1.86)	0.347
		CC	1(1.25)	N.C.	N.C.	N.C.	N.C.
		TC+CC	10(12.50)	1.76 (0.63-4.93)	0.285	0.57 (0.17-1.86)	0.347
				79			
ATG10	rs1864183	AA	60(75.79)	Reference		Reference	

		AG	18(22.78)	1.04 (0.41-2.63)	0.935	1.44 (0.66-3.15)	0.360
		GG	1(1.27)	N.C.	N.C.	N.C.	N.C.
		AG+GG	19(24.05)	1.61 (0.48-2.78)	0.739	1.44 (0.66-3.15)	0.360
				79			
		TT	54(68.35)	Reference		Reference	
ATG10	rs3734114	TC	22(27.85)	1.14 (0.49-2.70)	0.759	0.60 (0.26-1.39)	0.231
		CC	3(3.80)	N.C.	N.C.	N.C.	N.C.
		TC+CC	25(31.65)	1.07 (0.48-2.42)	0.867	0.70 (0.32-1.53)	0.370
				81			
		AA	35(43.21)	Reference		Reference	
ATG12	rs26532	AC	31(38.27)	1.47 (0.59-3.70)	0.412	0.82 (0.38-1.75)	0.605
		CC	15(18.52)	0.80 (0.27-2.33)	0.679	2.21 (0.82-5.97)	0.116
		AC+CC	46(56.79)	1.17 (0.53-2.54)	0.701	0.93 (0.47-1.84)	0.842
				77			
		GG	28(36.36)	Reference		Reference	
ATG12	rs26534	GA	38(49.35)	1.64 (0.65-4.11)	0.292	0.92 (0.46-1.84)	0.815
		AA	11(14.29)	1.06 (0.36-3.08)	0.923	1.12 (0.25-5.04)	0.886
		GA+AA	49(63.64)	1.24 (0.57-2.70)	0.585	0.89 (0.46-1.70)	0.719
				80			
		CC	31(38.75)	Reference		Reference	
ATG12	rs26538	CT	40(50.00)	1.43 (0.59-3.60)	0.447	0.81 (0.42-1.58)	0.539
		TT	9(11.25)	2.43 (0.68-8.63)	0.170	2.45 (0.54-11.00)	0.243
		CT+TT	49(61.25)	1.31 (0.61-2.84)	0.490	0.95 (0.50-1.82)	0.885
				80			
		TT	36(45.00)	Reference		Reference	
ATG16L1	rs2241880	TC	31(38.75)	1.59 (0.71-3.57)	0.262	1.59 (0.73-3.46)	0.247
		CC	13(16.25)	1.75 (0.55-5.54)	0.341	1.45 (0.53-3.98)	0.470
		TC+CC	44(55.00)	1.56 (0.73-3.35)	0.251	1.44 (0.74-2.81)	0.285
				81			
		CC	66(81.48)	Reference		Reference	
ATG16L2	rs11235604	CT	14(17.28)	1.74 (0.67-4.50)	0.256	1.83 (0.76-4.40)	0.180
		TT	1(1.23)	N.C.	N.C.	N.C.	N.C.

		CT+TT	15(18.51)	1.86 (0.75-4.59)	0.178	1.83 (0.76-4.40)	0.180
				80			
		AA	50(62.50)	Reference		Reference	
BECN	rs11552192	AT	28(35.00)	1.51 (0.67-3.44)	0.324	0.74 (0.36-1.53)	0.422
		TT	2(2.50)	N.C.	N.C.	N.C.	N.C.
		AT+TT	30(37.50)	1.65 (0.74-3.69)	0.223	0.82 (0.41-1.65)	0.585

Note: SNP, single nucleotide polymorphism; HR, hazard ratio; CI, confidence interval; N.C., not calculated.
Hazard ratios (HRs) and 95% confidence intervals (CIs) for the association between SNPs and geifitinib-resistance were estimated by Cox regression adjusted by sex, age, smoking status, ECGO and stages.

Full-length EMSA blots

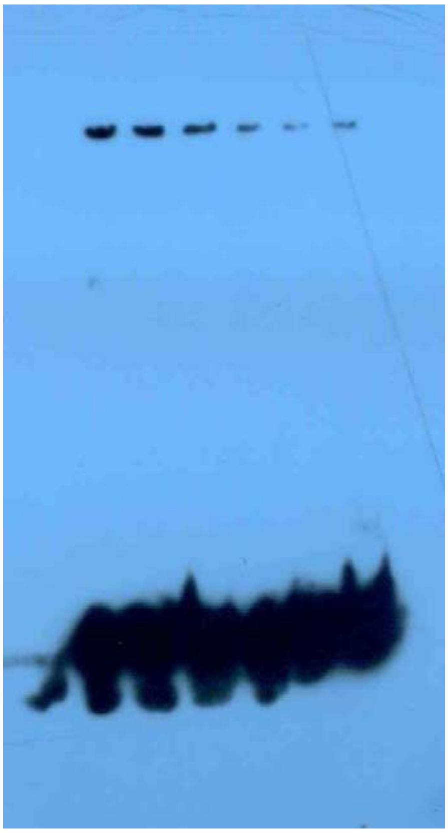

Supplement: Supplementary file 1 — Supplementary information [file 41598_2017_18165_MOESM1_ESM.pdf]
